# Citrate as a safe and effective alternative to heparin for catheter locking: a systematic review and meta-analysis of randomized controlled trials

**DOI:** 10.3389/fmed.2025.1530619

**Published:** 2025-02-26

**Authors:** Binbin Lai, Weixing Huang, Hui Yu, Tingting Chen, Yimen Gao, Wei Wang, Hua Luo

**Affiliations:** ^1^Department of Infection, Taizhou Hospital of Zhejiang Province Affiliated to Wenzhou Medical University, Taizhou, Zhejiang, China; ^2^Department of Nursing, Taizhou Hospital of Zhejiang Province Affiliated to Wenzhou Medical University, Taizhou, China; ^3^General Surgical Department, Taizhou Hospital of Zhejiang Province, Zhejiang University, Taizhou, China; ^4^Department of Nursing, Zhejiang University School of Medicine First Affiliated Hospital, Hangzhou, China; ^5^Department of Hematology, Taizhou Hospital of Zhejiang Province Affiliated to Wenzhou Medical University, Taizhou, China; ^6^Department of Orthopedics, Taizhou Hospital of Zhejiang Province Affiliated to Wenzhou Medical University, Taizhou, Zhejiang, China; ^7^Department of Nursing, Liaoning Technical University of Vocational, Jinzhou, Liaoning, China

**Keywords:** heparin, citrate, locking solution, catheter-related bloodstream infection, adverse event

## Abstract

**Background:**

Consensus on the use of citrate vs.heparin for catheter locking remains elusive, with ongoing controversy. This meta-analysis investigates the efficacy and safety of citrate lock solutions compared to heparin lock solutions in preventing catheter-related complications.

**Methods:**

The review process was conducted according to the Preferred Reporting Items for Systematic Reviews and Meta-Analyses (PRISMA) guidelines. Two independent reviewers conducted literature searches based on preferred reporting items from systematic reviews and meta-analyses. PubMed, EMBASE, Medline, and the Cochrane Library were searched for studies comparing citrate and heparin in patients with catheter. Catheter-related bloodstream infection (CRBSI), catheter-related infection (CRI), exit-site infection (ESI), and adverse events were analyzed.

**Results:**

The meta-analysis included 17 randomized controlled trials (RCTs), encompassing 247,431 catheter-days, with 128,904 in the citrate group, and 118,527 in the heparin group. Citrate lock solutions significantly reduced the incidence of CRBSI compared to heparin (RR: 0.48, 95% CI: 0.31–0.73), particularly when combined with antibiotics or used at low concentrations. No significant differences were observed between the groups for CRI, ESI, catheter dysfunction, or local bleeding. Subgroup and sensitivity analyses addressed heterogeneity, confirming the robustness of the primary findings.

**Conclusions:**

Citrate lock solutions effectively prevent CRBSI without increasing systemic coagulation dysfunction or bleeding risk. Citrate lock solutions are a safe and effective alternative to heparin, especially when combined with antibiotics.

**Systematic review registration:**

https://www.crd.york.ac.uk/prospero/display_record.php?ID=CRD42024562511.

## Introduction

Vascular catheter pathways are widely used in clinical settings, particularly in intensive care units, chemotherapy, hemodialysis, and long-term parenteral nutrition (1, 2). Currently, the most commonly used devices include central venous catheters (CVCs), non-tunneled-uncuffed catheters (NTCs), tunneled-cuffed catheters (TCCs), peripherally inserted central catheters (PICCs), and totally implantable venous access ports (TIVAPs) to address the needs of patients requiring long-term intravenous infusions or hemodialysis (3, 4).

Catheter-related bloodstream infection (CRBSI) is one of the most serious complications associated with vascular catheters. The incidence of CRBSI is influenced by factors such as catheter type (5), patient conditions (including advanced age, diabetes, hypoproteinemia, and prolonged steroid or immunosuppressive therapy) (6–8), operator experience (9), and the duration of catheter placement (10, 11). Among these, ICU patients with implanted CVCs and hemodialysis patients with NTC or TCC catheters are most commonly affected, with hemodialysis patients using NTCs being more susceptible to CRBSI compared to those using TCCs (12). The occurrence of CRBSI not only extends the patient's hospital stay but also increases medical costs and mortality rates.

Ensuring catheter patency through appropriate locking is crucial for the effective prevention of thrombosis and CRBSI during the use of central venous catheters. Therefore, exploring effective catheter locking methods to prevent CRBSI caused by intravascular catheters holds significant clinical importance. Citrate, as a local anticoagulant, chelates serum calcium ions without affecting systemic coagulation function and also possesses antibacterial properties. Its use in catheter locking is becoming increasingly widespread.

However, there is no consensus on the superiority of citrate vs. heparin locking solutions for catheter locking, and some controversy remains. Thus, this study aims to compare the efficacy of citrate and citrate lock solutions combined with antibiotics, vs. heparin locking solutions, in preventing catheter-related complications through a meta-analysis, providing a reference for clinical practice.

## Methods

This meta-analysis was performed according to the Preferred Reporting Items for Systematic Reviews and Meta-Analyses (PRISMA) guidelines and has been reported in line with the AMSTAR (Assessing the methodological quality of systematic reviews) Guideline (13, 14). The protocol for this meta-analysis was registered on PROSPERO (registration ID: CRD42024562511).

### Inclusion criteria

(1) Clinical studies comparing citrate and heparin lock solutions in the prevention of catheter-related complications;
(2) Randomized controlled trials (RCT);
(3) Full text available;
(4) Participants older than 18 years.

### Exclusion criteria

(1) Studies if they were letters, case reports, reviews, animal trials, or republished studies;
(2) Studies with incomplete or missing data relevant to the analysis will be excluded;
(3) Cohort studies, and case-control studies;
(4) CVCs used for chemotherapy.

### Outcomes

The primary outcome was the CRBSI. The second outcome included Catheter-related infection (CRI), exit-site infection (ESI), and adverse events.

### Search strategy

Two of the authors performed the search in PubMed, EMBASE, Medline, and the Cochrane Central Register of Controlled Trials from the inception dates to July 2024, using the keywords “citrate”, “heparin”, “locking solution”, and “infection”. No language restrictions were applied during the search.

### Data collection process

Two investigators used a standard data extraction form to extract all related data from selected trials independently. Data extracted included the first author's name, year of publication, country, participants, CVC type, patients setting, locking solution, sample size, catheter-days, sex, age, and related outcomes. Disagreements were resolved by consensus.

### Assessment of risk of bias and quality of evidence

Two researchers independently assessed the quality of RCTs using the Cochrane risk-of-bias criteria (15). When they consider their methods, researchers decide whether those assessing the risk of bias will be blinded to the authors' names, institutions, journals, and study results. Disagreements were resolved by consensus.

### Data synthesis

The meta-analysis used Stata software (version 17; StataCorp, 2021). Heterogeneity was assessed *via* the *Q*-test and calculation of the *I*^2^ value. Our analysis employed the random effects model. Relative risk (RR) with corresponding 95% confidence intervals (CI) were used for count outcome assessment. Statistical significance was denoted by a *P-*value below 0.05. When dealing with multiple correlated comparisons in the same experiment, following the guidance provided by the Cochrane Handbook, which combine groups to create a single pair-wise comparison (16).

### Sensitivity analyses

We performed a sensitivity analysis by excluding individually trials.

## Results

### Eligible studies

**Figure 1:**
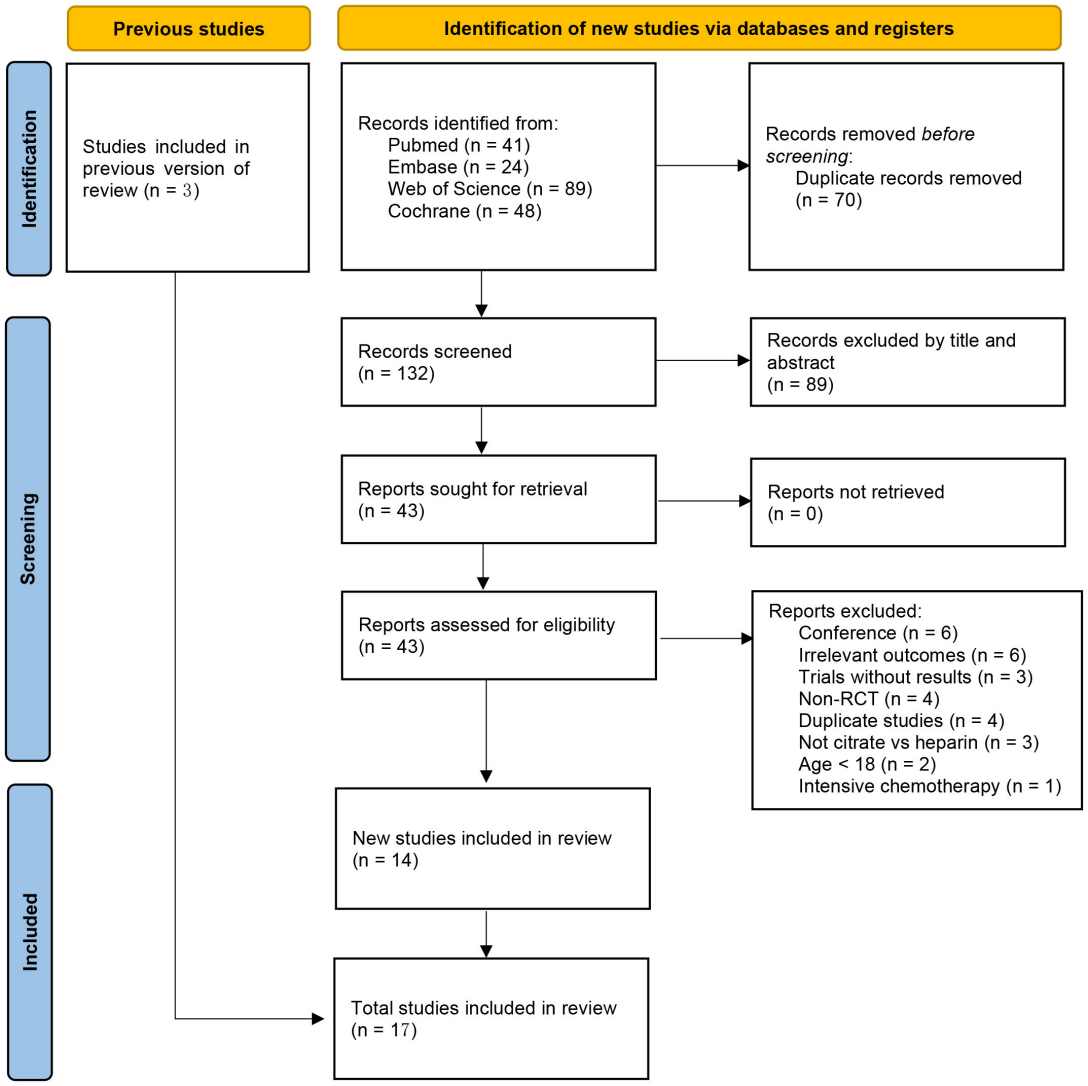
Flow diagram for search and selection of included studies.

Initially, a total of 202 relevant articles were identified. After removing 70 duplicate articles, 132 articles remained. Screening the titles and abstracts of these articles led to the exclusion of 89 irrelevant articles. The full texts of the remaining 43 articles were reviewed, resulting in the exclusion of 29 studies. These exclusions included 6 conference abstracts, 6 studies with outcomes not relevant to our research, 3 studies with no results, 4 non-randomized controlled trials, 4 duplicate studies, 3 studies not comparing sodium citrate vs. heparin, 2 studies involving children, and 1 study involving patients with hematological malignancies undergoing intensive chemotherapy. Three studies from previous research were included, bringing a total of 17 trials included in our meta-analysis (17–33). The search detail was shown in Figure 1.

### Quality of trials

**Figure 2:**
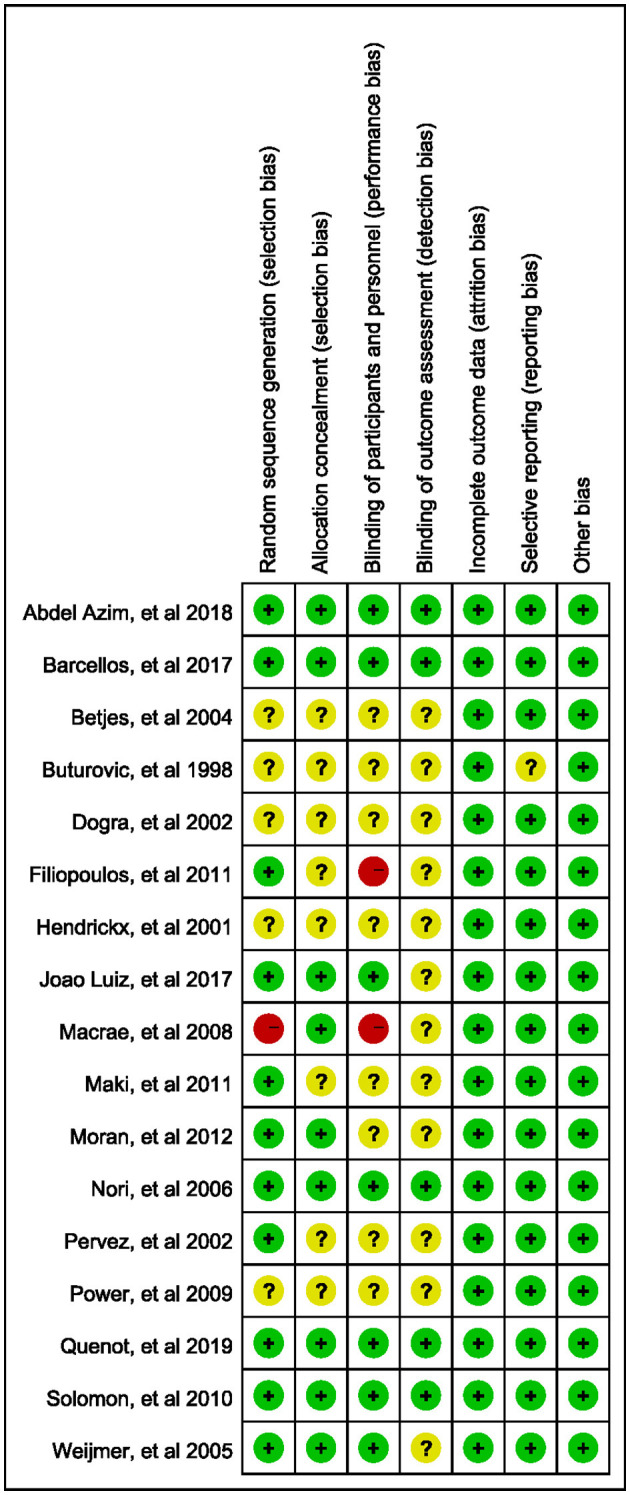
Risk of bias summary.

The quality of the included studies was assessed using the Cochrane risk-of-bias tool. Seven studies were of high quality, eight had moderate quality, and two had low quality. The primary sources of bias were the blinding of participants and personnel (Figure 2).

### CRBSI

**Figure 3:**
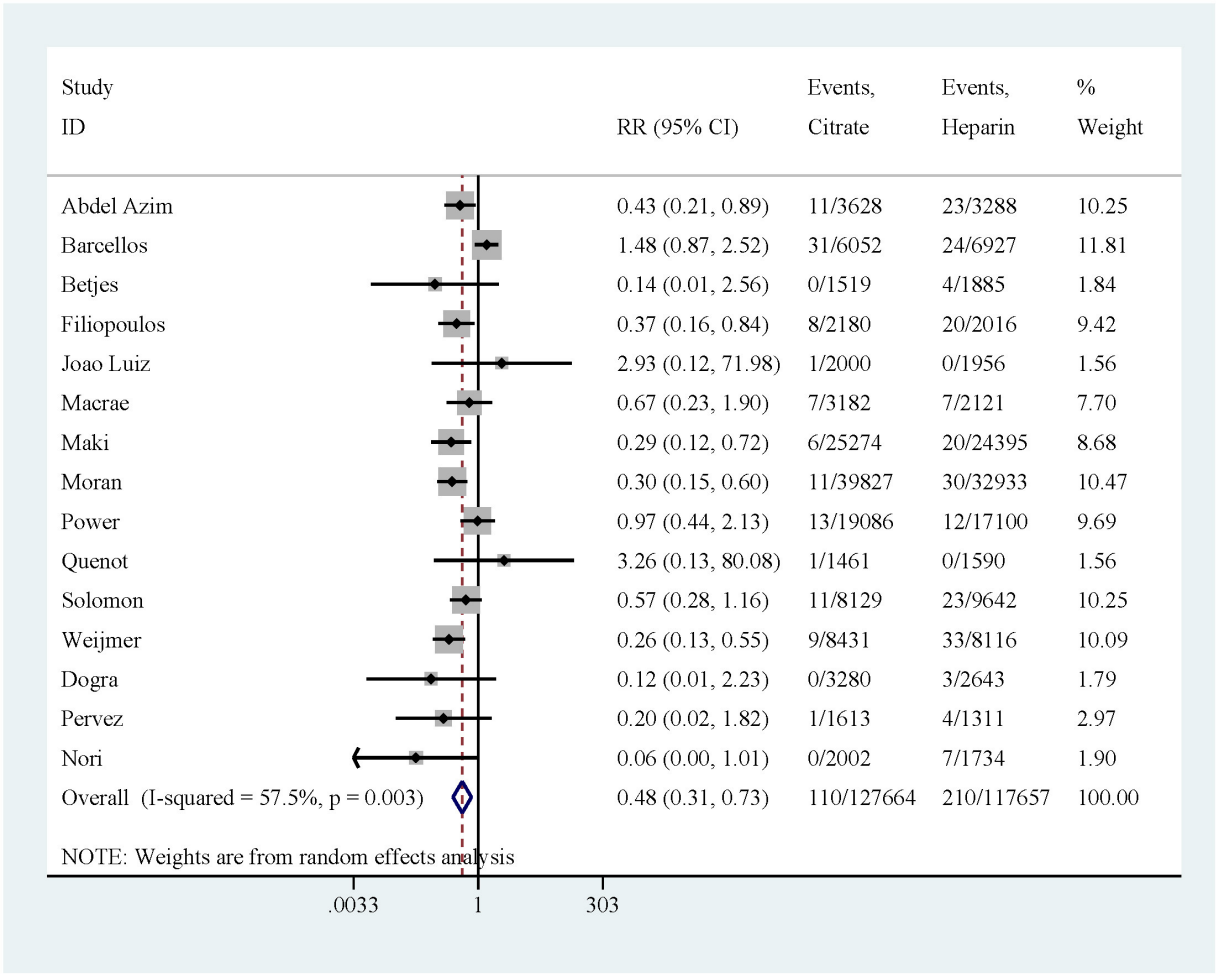
Forest plot for catheter-related bloodstream infection.

**Figure 4:**
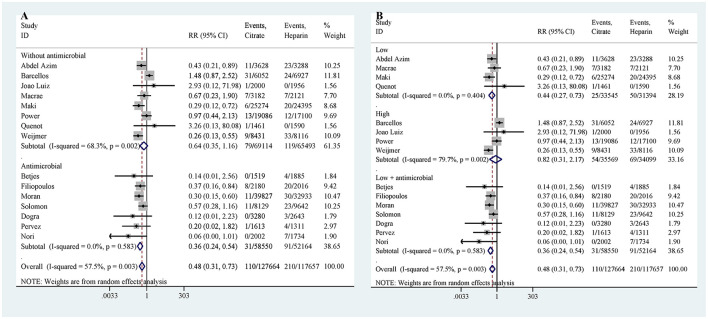
Subgroup analysis for catheter-related bloodstream infection [Citrate solutions with and without antibiotics **(A)**; Low-, low+antimicrobial, and high-concentration citrate solutions **(B)**].

A total of 15 studies reported on CRBSI. The results indicated that the risk of CRBSI was significantly lower in the citrate lock solution group compared to the heparin lock solution group (RR: 0.48, 95% CI: 0.31–0.73, *P* = 0.001, *I*^2^ = 57.5%; Figure 3). Moderate heterogeneity was observed in the results. To address this, we considered whether the addition of antibiotics to citrate solutions could be a source of heterogeneity and conducted a subgroup analysis based on the presence of antibiotics in the citrate solution. The subgroup analysis revealed that citrate solutions without antibiotics did not significantly reduce the incidence of CRBSI (RR: 0.64, 95% CI: 0.35–1.16, *P* = 0.141, *I*^2^ = 68.3%; Figure 4A). In contrast, citrate solutions containing antibiotics significantly reduced the incidence of CRBSI (RR: 0.36, 95% CI: 0.24–0.54, *P* < 0.001, *I*^2^ = 0%; Figure 4A). Additionally, we conducted a subgroup analysis based on the concentration of citrate in the solution. For citrate combined with antibiotics, all solutions were of low concentration, and the results were consistent with those mentioned above. For citrate without antibiotics, the analysis was divided into low- and high-concentration groups. In the low-concentration subgroup, citrate demonstrated a significant reduction in CRBSI incidence compared to heparin (RR: 0.44, 95% CI: 0.27–0.73, *P* = 0.001, *I*^2^ = 0%; Figure 4B). However, in the high-concentration subgroup, no significant difference was observed between the two groups (RR: 0.82, 95% CI: 0.31–2.17, *P* = 0.682, *I*^2^ = 79.7%; Figure 4B). Notably, the high-concentration subgroup exhibited substantial heterogeneity, which resolved (*I*^2^ = 0%) upon excluding the study by Weijmer et al. (33), with the conclusion remaining unchanged.

### CRI

**Figure 5:**
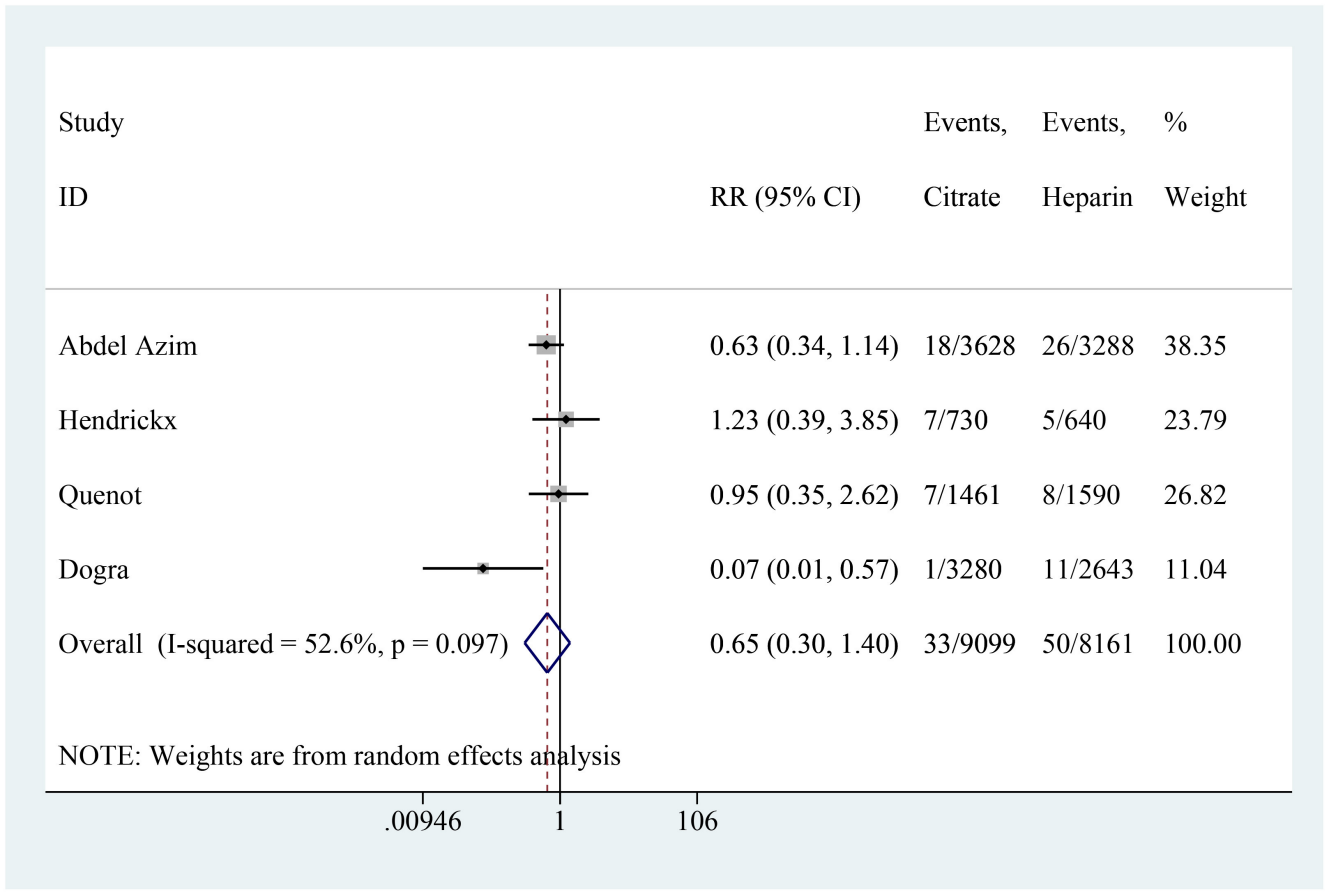
Forest plot for catheter-related infection.

**Figure 6:**
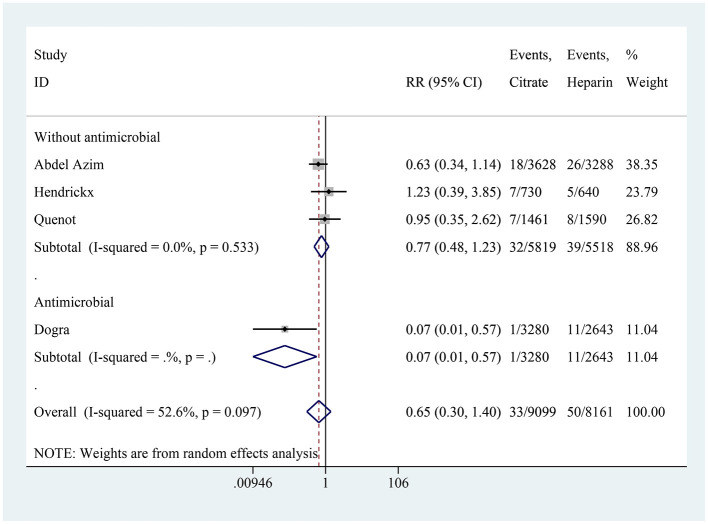
Subgroup analysis for catheter-related infection.

Four studies reported on the incidence of CRI, with the citrate group comprising 9,099 catheter-days and the heparin group comprising 8,161 catheter-days. The aggregated results showed no difference in the incidence of CRI between the citrate and heparin lock solution groups (RR: 0.65, 95% CI: 0.30–1.40, *P* = 0.272, *I*^2^ = 52.6%; Figure 5). Substantial heterogeneity was observed in the studies. Upon analyzing the included literature, we found that four studies did not use antibiotic-containing citrate solutions, whereas the study by Dogra et al. (21) used an antibiotic-containing citrate solution. To further explore the impact of antibiotics, a subgroup analysis was performed based on the presence or absence of antibiotics. In the subgroup without antibiotics, no significant difference was observed between the two groups (RR: 0.77, 95% CI: 0.48–1.23, *I*^2^ = 0%; Figure 6). However, in the subgroup with antibiotics, citrate was found to significantly reduce the incidence of CRI (RR: 0.07, 95% CI: 0.01–0.57; Figure 6).

### ESI

**Figure 7:**
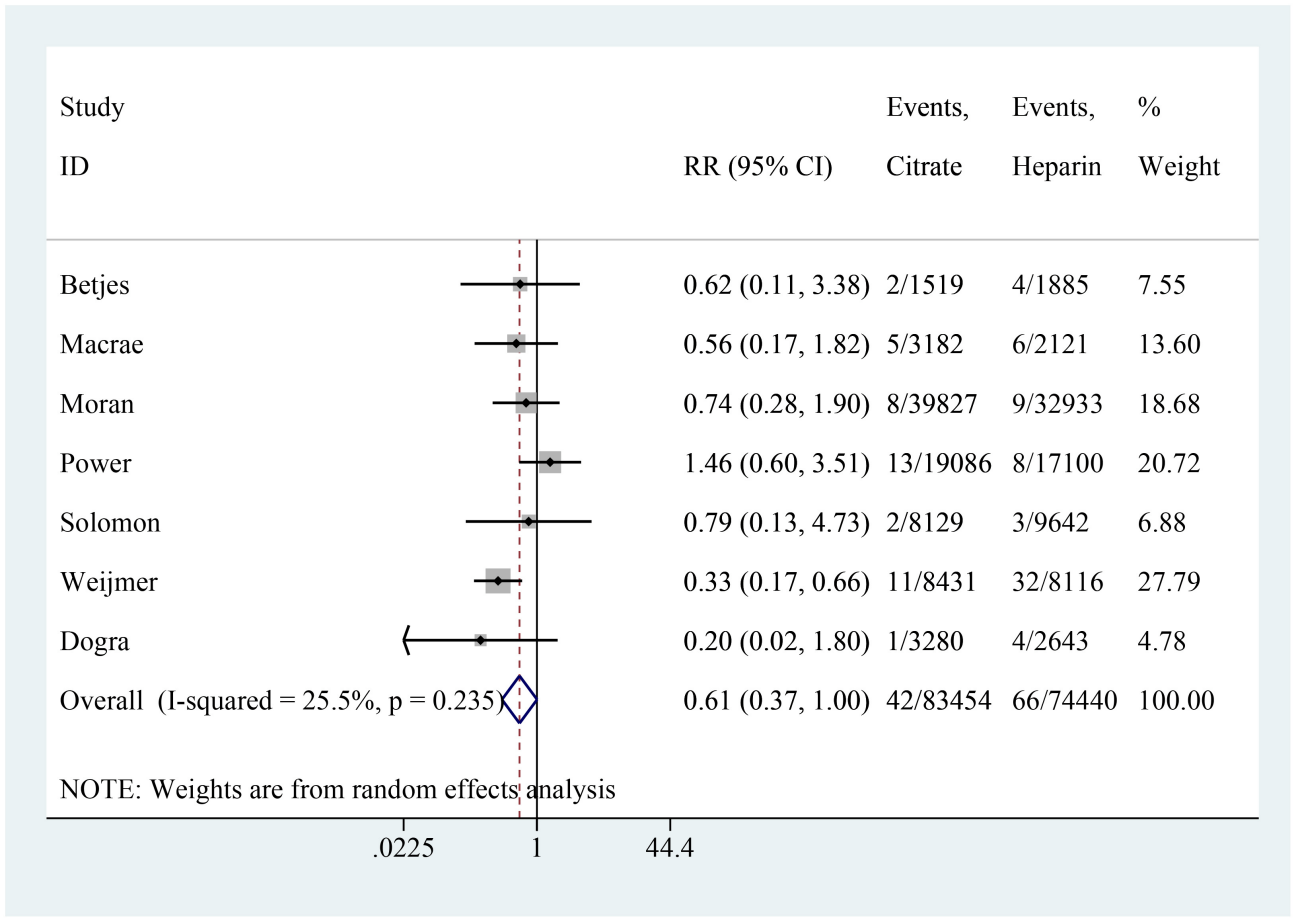
Forest plot for exit-site infection.

**Figure 8:**
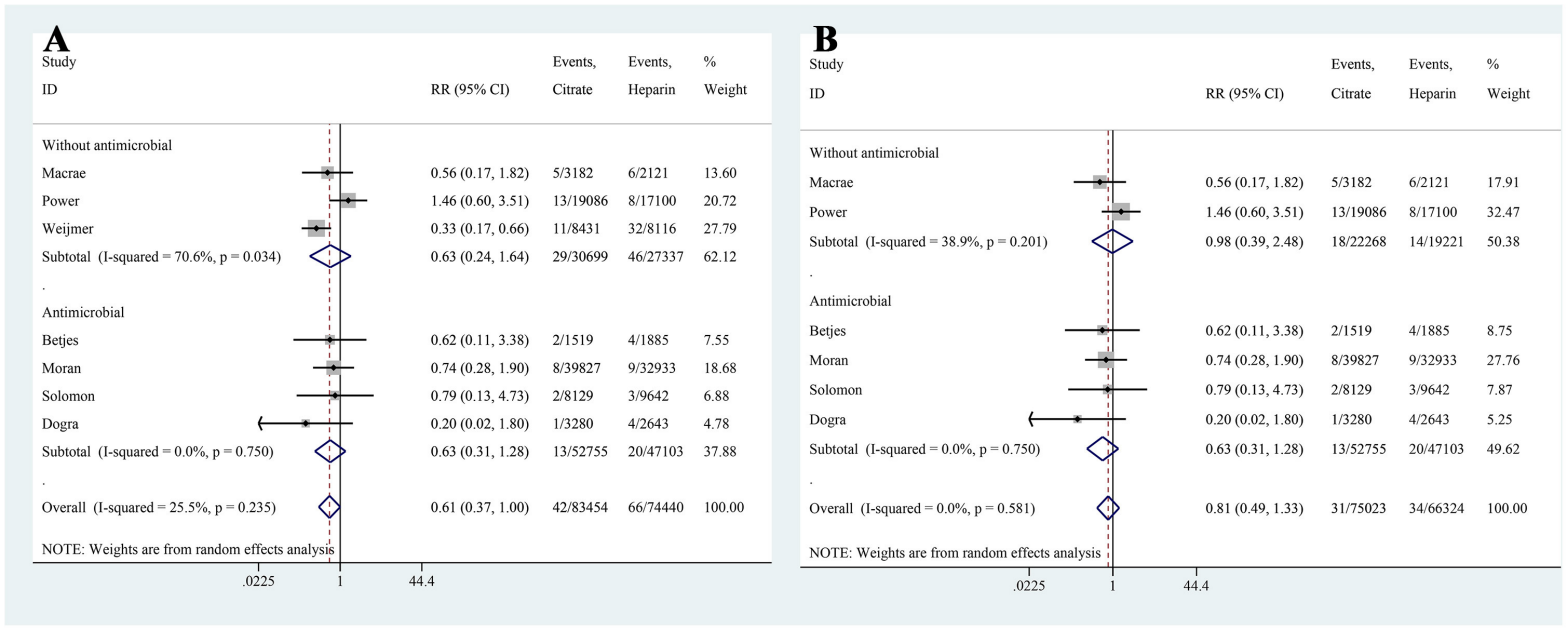
Subgroup analysis for exit-site infection [All trials **(A)**; After excluding Weijmer et al.'s (33) trial **(B)**].

Seven studies reported on the incidence of ESI, with the citrate group comprising 83,454 catheter-days and the heparin group comprising 74,440 catheter-days. The pooled analysis indicated no significant difference in ESI incidence between the two groups (RR: 0.61, 95% CI: 0.37–1.00, *P* = 0.052, *I*^2^ = 25.5%; Figure 7). A subgroup analysis was performed based on the use of antibiotics, and both subgroups showed no difference in ESI incidence between the two groups (Figure 8A). In the subgroup without antibiotics, high heterogeneity was observed (*I*^2^ = 70.6%). After excluding Weijmer et al.'s study (33), the heterogeneity decreased substantially (*I*^2^ = 38.9%; Figure 8B), with the conclusion remaining unchanged.

### Catheter dysfunction

**Figure 9:**
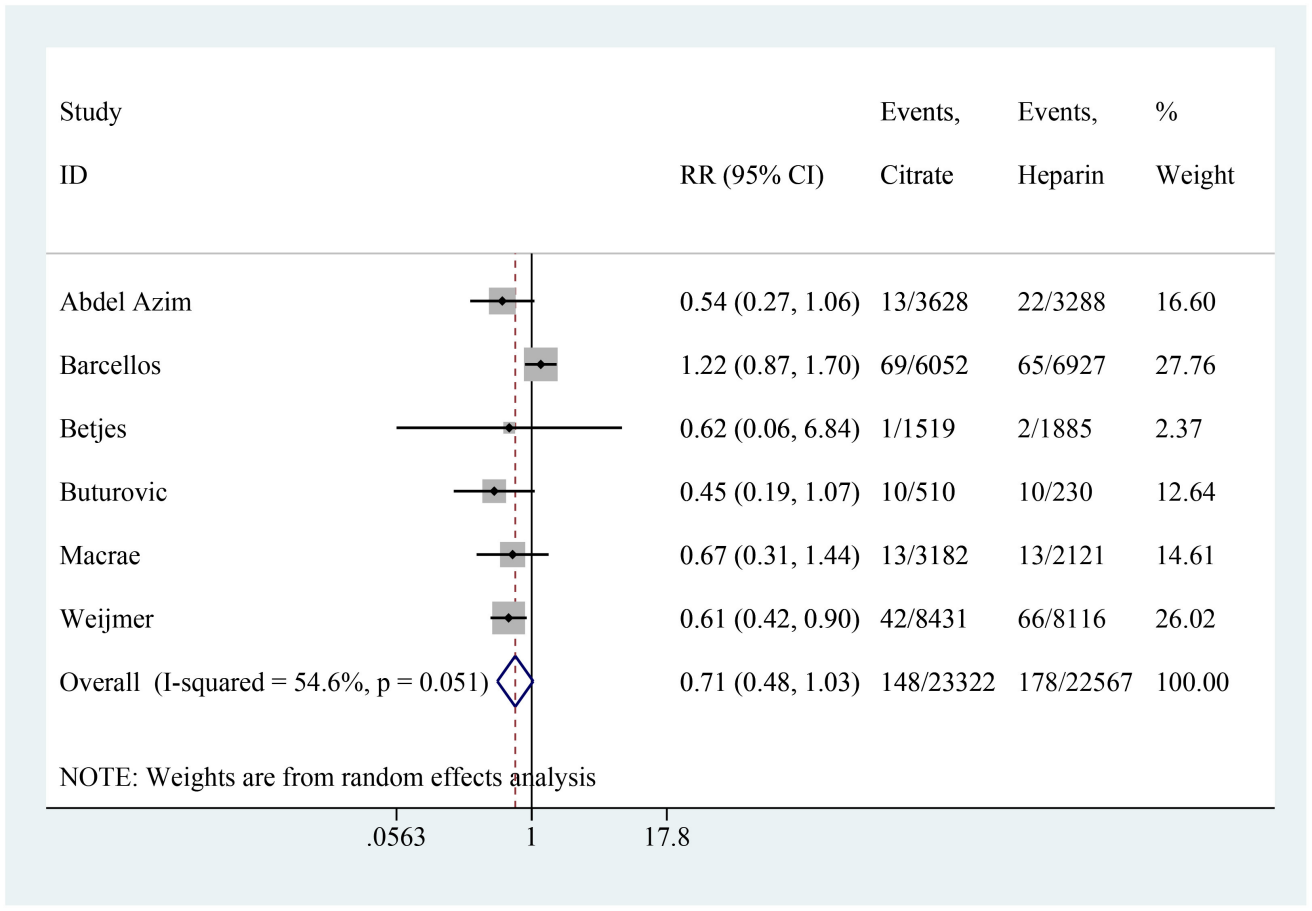
Forest plot for catheter dysfunction.

Six studies reported on catheter dysfunction, with the citrate group comprising 23,322 catheter-days and the heparin group comprising 22,567 catheter-days. The pooled results showed no significant difference in catheter dysfunction between the citrate and heparin lock solution groups (RR: 0.71, 95% CI: 0.48–1.03, *P* = 0.074, *I*^2^ = 54.6%; Figure 9).

### Local bleeding

**Figure 10:**
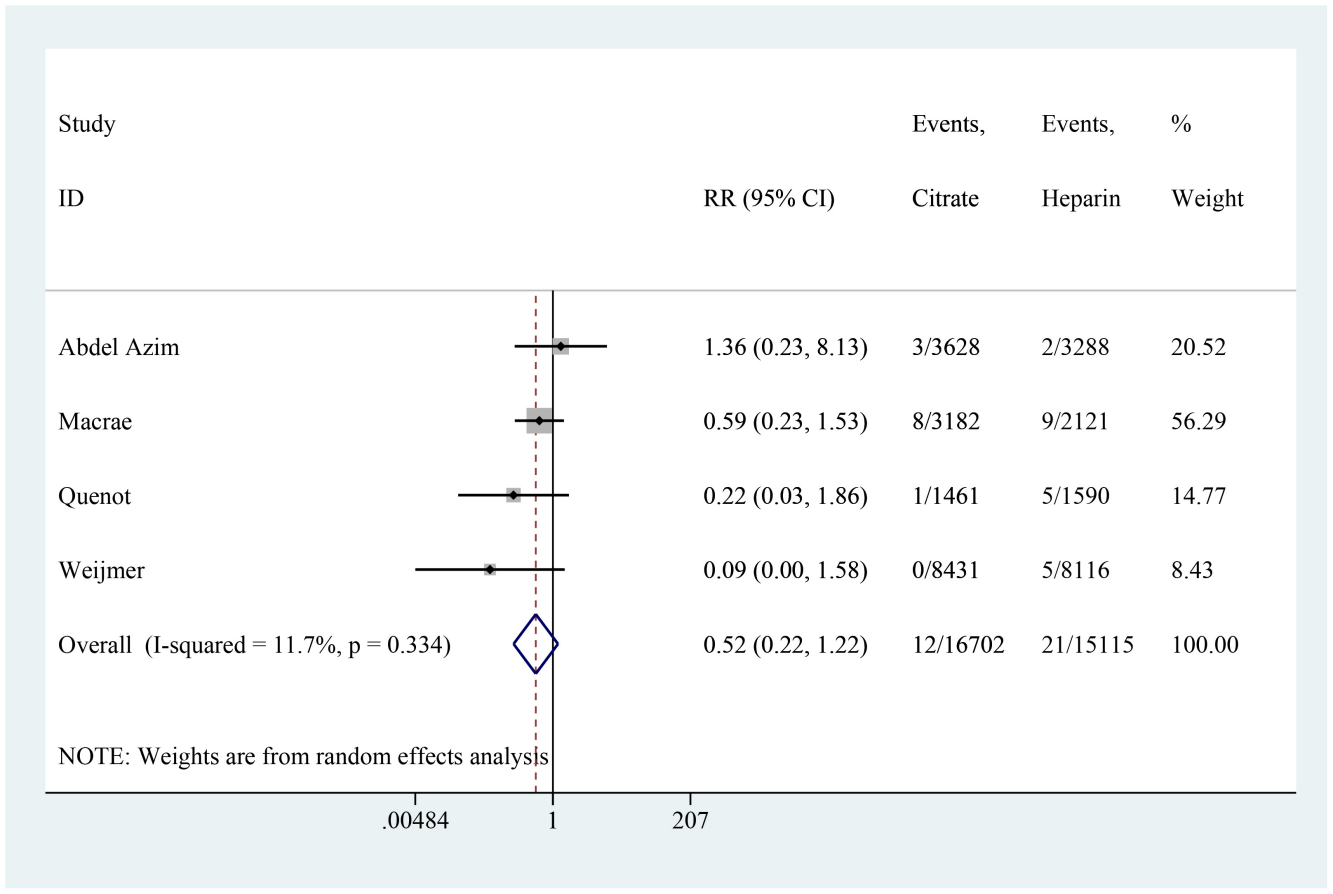
Forest plot for local bleeding.

Four studies, encompassing a total of 31,817 catheter-days, reported on local bleeding. The combined results showed no significant difference in the incidence of local bleeding between the citrate and heparin lock solution groups (RR: 0.52, 95% CI: 0.22–1.22, *P* = 0.132, *I*^2^ = 11.7%; Figure 10).

### Meta-regression

Considering that age differences could be a potential source of heterogeneity, we conducted a meta-regression with age as a covariate for CRBSI, CRI, and ESI. The results showed no statistically significant association between age and these outcomes (*P* > 0.05 for all) (Table 1).

**Table 1:** Meta-regression analysis of age as a covariate on CRBSI, CRI, and ESI.

**Outcomes**	**Covariate**	**Coefficient**	**SE**	***Z***	***P***	**95% CI**
CRBSI	Age	−0.009	0.029	−0.32	0.75	−0.065, 0.047
CRI	Age	0.024	0.025	0.97	0.334	−0.025, 0.073
ESI	Age	0.026	0.082	0.32	0.75	−0.134, 0.186

CRBSI, Catheter-related bloodstream infection; CRI, Catheter-related infection; ESI, exit-site infection.

### Sensitivity analysis

The remaining studies were combined when any individual study was excluded. No particular study had a significant impact on the results.

### Publication of bias

**Figure 11:**
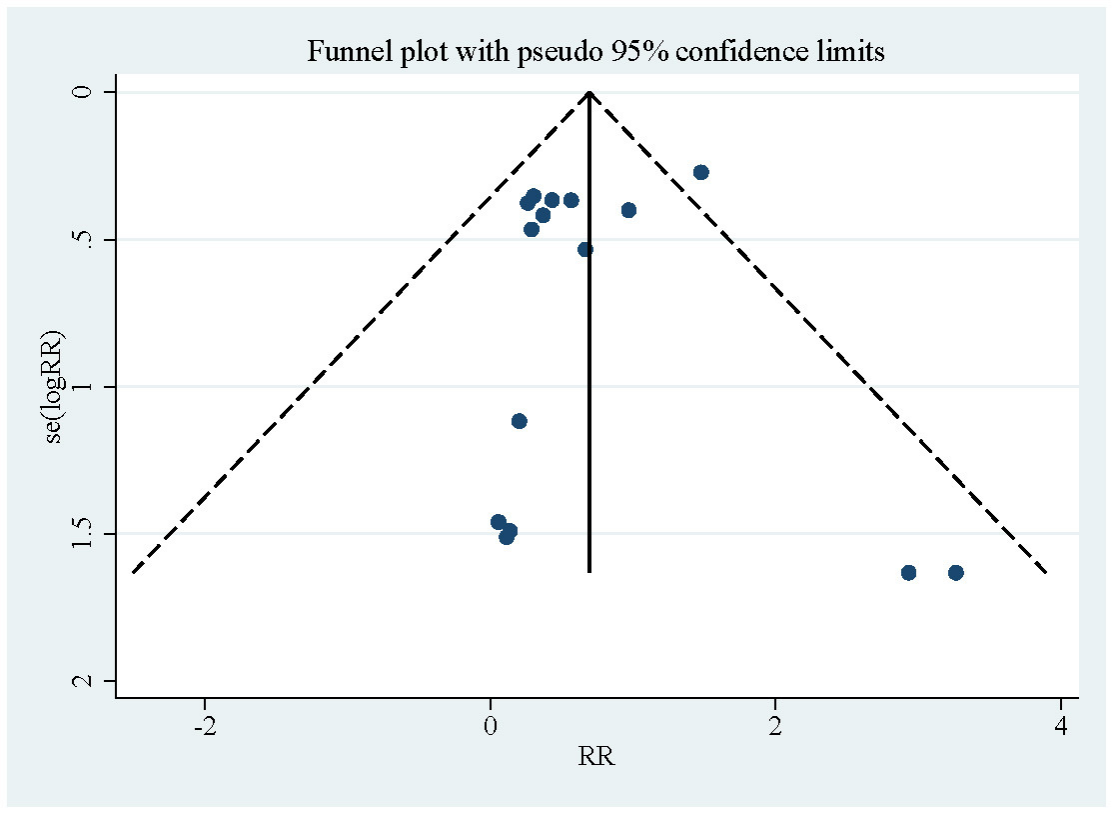
Funnel plot of the included studies in this meta-analysis for catheter-related bloodstream infection.

Figure 11 shows that small sample studies may be the leading cause of bias.

## Discussion

This meta-analysis included 17 RCTs, comprising 128,904 catheter-days in the citrate group and 118,527 catheter-days in the heparin group. The combined results showed that the citrate group had a significantly lower incidence of CRBSI compared to the heparin group. Considering the substantial heterogeneity of the results, a subgroup analysis was conducted based on whether antibiotics were included in the citrate solution. The results indicated that citrate solutions with antibiotics significantly reduced the incidence of CRBSI, whereas, there was no difference between citrate without antibiotics and heparin lock solutions in CRBSI incidence. Additionally, subgroup analysis based on citrate concentration indicated that low-concentration citrate, with or without antibiotics, significantly reduced CRBSI incidence. In contrast, high-concentration citrate showed no difference between the two groups. There was no difference in the incidence of CRI between the two groups, primarily because the studies included used citrate lock solutions without antibiotics. In the study by Dogra et al., the use of antibiotic-containing citrate lock solution significantly reduced the incidence of CRI compared to heparin lock solution. Citrate showed no significant difference in reducing the incidence of ESI compared to heparin. Sensitivity analysis identified the study by Wenjmer et al. as the primary source of heterogeneity (33). After excluding this study, heterogeneity was significantly reduced, and the conclusion remained unchanged. A possible explanation for this finding is that the study exclusively included patients with newly inserted, well-positioned hemodialysis catheters expected to be used for more than 1 week. To further explore potential sources of heterogeneity, a meta-regression analysis was conducted using age as a covariate for CRBSI, CRI, and ESI. The results showed no statistically significant association between age and these outcomes. For CRBSI, the coefficient was −0.009 (95% CI: −0.065 to 0.047, *P* = 0.621), indicating a minimal and non-significant negative correlation with age. For CRI, the coefficient was 0.024 (95% CI: −0.025 to 0.073, *P* = 0.334), suggesting a slight, non-significant positive association. Similarly, for ESI, the coefficient was 0.026 (95% CI: −0.134 to 0.186, *P* = 0.750), reflecting a negligible and non-significant positive association. These findings suggest that age is unlikely to be a meaningful contributor to the observed heterogeneity, further supporting the robustness of the conclusions. There were no significant differences between the two groups in the incidence of catheter dysfunction and local bleeding. Besides, we attempted to explore potential factors influencing CRBSI, including patient characteristics such as age, comorbidities, and nutritional status. However, after reviewing the included studies, we found that none performed subgroup analyses based on these factors. Although patient conditions such as controlling blood glucose levels in diabetes and enhancing nutritional support may play a significant role in reducing infection risks, the available data did not allow for a detailed subgroup analysis in this context. Therefore, we emphasize the importance of optimizing the overall clinical condition of patients, particularly in terms of managing underlying health conditions, to reduce CRBSI incidence.

Biofilms are complex microbial communities that adhere to the surface of catheters and are a major cause of CRBSIs (34). Citrate can disrupt biofilm formation, thereby reducing the incidence of CRBSI, especially when combined with antibiotics (35, 36). However, in the absence of antimicrobial agents, citrate alone may be insufficient to disrupt established biofilms, resulting in no significant difference compared to heparin (37). CRBSIs are typically caused by contamination within the catheter or by skin microorganisms migrating along the catheter (34). The antimicrobial properties of citrate are more effective within the lumen where the solution is in direct contact with the biofilm (38). On the other hand, puncture occurs at the catheter exit site, where the skin barrier is breached. The chelating action of citrate may help reduce the microbial load at the exit site, thus lowering the incidence of ESI even without added antibiotics. Heparin sodium, commonly used as a locking solution, may cause systemic coagulation dysfunction and bleeding, including complications such as heparin-induced thrombocytopenia, which limits its clinical application to some extent (39, 40). Citrate binds to calcium ions in the blood and breaks down into carbon dioxide and other products, providing anticoagulation without causing systemic coagulation dysfunction or increasing bleeding risk, and it possesses inherent antibacterial activity (41). Our results indicate that citrate significantly reduces the incidence of CRBSI and that citrate without antibiotics is as effective as heparin in preventing ESI and CRI without increasing the occurrence of related complications.

Previous studies (42, 43) have shown that the combined use of citrate and antibiotics (such as gentamicin, taurolidine, EDTA, etc.) can reduce the incidence of sepsis and shorten treatment duration. This meta-analysis also suggests that using citrate locks can better prevent CRBSI. Subgroup analysis shows that the combination of citrate and antibiotic locks effectively prevents CRBI, while the use of citrate alone does not show a statistically significant difference compared to heparin, consistent with our previous research. Our study differs significantly from previous research by including all patients with indwelling catheters, rather than limiting the analysis to hemodialysis patients. This broader inclusion criterion allows for a more comprehensive evaluation of the effectiveness of citrate lock solutions across different patient populations. Previous studies have primarily focused on hemodialysis patients, often excluding other critical groups such as ICU patients, and those requiring long-term parenteral nutrition. By encompassing a wider range of patients, our study provides a more generalized understanding of the benefits of citrate lock solutions. This inclusive approach is particularly important because it reflects real-world clinical settings where various types of catheters are used for different medical purposes. Furthermore, the broader inclusion of various patient populations allows us to observe the safety profile of citrate lock solutions more accurately. Our results indicate that citrate does not cause systemic coagulation dysfunction or increase the risk of bleeding, making it a safer alternative to heparin, especially for patients who are already at a higher risk of bleeding complications.

## Limitation

Although this study included only RCTs, several limitations should be noted. First, the follow-up periods varied among the studies, which may contribute to heterogeneity in the results. Second, the inclusion criteria differed, and variations in citrate concentration and the use of antibiotics also increased heterogeneity. Third, the definitions of catheter malfunction listed in Table 2 were not completely consistent, which may cause substantial heterogeneity. Forth, this study is the unable to conduct subgroup analyses based on patient characteristics. Despite these factors potentially influencing the outcomes, the included studies did not perform such subgroup analyses, and therefore, we were unable to assess their direct impact. This is an area for future research, where stratifying by patient condition could provide more tailored recommendations.

**Table 2:** Characteristics of included studies.

**Study**	**Country**	**Participants**	**CVC type**	**Patients setting**	**Treatment group**	**Control group**	**No. of subjects**		**Catheter-days**		**Sex (female)**		**Age**		**Outcomes**
							**Citrate**	**Heparin**	**Citrate**	**Heparin**	**Citrate**	**Heparin**	**Citrate**	**Heparin**	
Abdel Azim et al. (17)	Egypt	Hemodialysis patients	NS	Maintenance hemodialysis	4% citrate	Heparin 5,000 U/mL	105	105	3,628	3,288	47	40	51.33 ± 11.2	51.74 ± 13.1	CRBSI, CRI, catheter dysfunction, thrombosis, bleeding
Barcellos et al. (18)	Brazil	Hemodialysis patients	NS	Maintenance hemodialysis	30% trisodium citrate	Heparin 5,000 U/mL	231	233	6,052	6,927	121	116	58.61 ± 17.14	57.44 ± 18.27	CRBSI, catheter dysfunction, adverse event, death
Betjes et al. (19)	Netherlands	Hemodialysis patients	Non-tunneled	Maintenance hemodialysis	4% citrate + 1.35% taurolidine	Heparin 5,000 U/mL	37	39	1,519	1885	16	15	58.3 ± 16.3	50.3 ± 20.4	CRBSI, catheter dysfunction, adverse event, ESI
Buturovic et al. (20)	USA	Hemodialysis patients	NS	Maintenance hemodialysis	4% trisodium citrate	Heparin 5,000 U/mL	10	10	510	230	NA	NA	NA	NA	Catheter dysfunction
Filiopoulos et al. (22)	Greece	Hemodialysis patients	Uncuffed	Maintenance hemodialysis	4% citrate + 1.35% taurolidine	Heparin 5,000 U/mL	59	58	2,180	2,016	26	28	75 (36–95)	70 (42–84)	CRBSI, thrombosis, adverse effects
Hendrickx et al. (23)	Belgium	Hemodialysis patients	Tunneled	Maintenance hemodialysis	5% trisodium citrate	Heparin 5,000 U/mL	10	9	730	640	6	5	74.6	71.4	CRI
Joao Luiz et al. (24)	Brazil	Hemodialysis patients	NS	Maintenance hemodialysis	30% trisodium citrate	Heparin 1,000 IU/mL	25	25	2,000	1,956	NA	NA	NA	NA	CRBSI, adverse events
Macrae et al. (25)	Canada	Hemodialysis patients	Cuffed	Maintenance hemodialysis	4% citrate	Heparin 5,000 U/mL	32	29	3,182	2,121	11	15	63 ± 16	69 ± 15	CRBSI, catheter dysfunction, bleeding, ESI, cost
Maki et al. (26)	USA	Hemodialysis patients	Cuffed and tunneled	Maintenance hemodialysis	7.0% citrate + 0.05% methylene blue + 0.15% methylparaben + 0.015% propylparaben	Heparin 5,000 U/mL	201	206	25,274	24,395	103	100	62.2 ± 15.4	61.7 ± 15.2	CRBSI, adverse events
Moran et al. (27)	USA	Hemodialysis patients	Cuffed and tunneled	Maintenance hemodialysis	4% citrate + 20 g/mL of gentamicin	Heparin 1,000 U/mL	155	148	39,827	32,933	79	67	63.4 ± 15.6	62.8 ± 16.8	CRBSI, catheter clotting
Power et al. (30)	UK	Hemodialysis patients	Cuffed	Maintenance hemodialysis	46.7% citrate	Heparin 5,000 U/mL	132	100	19,086	17,100	59	41	63 ± 14	62 ± 13	CRBSI, adverse events
Quenot et al. (31)	France	Critically ill patients	Tunneled	Critically ill patients	4% trisodium citrate	Heparin 5,000 U/mL	199	197	1,461	1,590	72	74	69.4 ± 13.4	69.5 ± 12.9	CRBSI, bleeding, thrombosis, death
Solomon et al. (32)	England	Hemodialysis patients	Tunneled cuffed	Maintenance hemodialysis	4% citrate + 1.35% taurolidine	Heparin 5,000 U/mL	53	54	8,129	9,642	27	13	59.8 ± 14.7	56.7 ± 17.4	CRBSI, ESI, adverse events
Weijmer et al. (33)	Belgium	Hemodialysis Patients	Tunneled cuffed and untunneled uncuffed	Maintenance hemodialysis	30% trisodium citrate	Heparin 5,000 U/mL	148	143	8,431	8,116	87	87	61.6 ± 14.8	61.3 ± 16	CRBSI, bleeding, adverse events
Dogra et al. (21)	Australia.	Hemodialysis patients	Tunneled cuffed	Maintenance hemodialysis	1.04% citrate + 26.7 mg/mL gentamicin	Heparin 5,000 U/mL	42	37	3,280	2,643	18	11	55.7 ± 2.5	59.3 ± 2.1	CRBSI, ESI, CRI
Pervez et al. (29)	USA	Tunnel catheter placement patients	Tunneled cuffed	Maintenance hemodialysis	4.6% citrate + 18.2 mg/mL gentamicin	Heparin 1,000 U/mL	14	22	1,613	1,311	4	12	53.7 ± 4.0	47.6 ± 3.3	CRBSI, thrombosis
Nori et al. (28)	USA	Hemodialysis patients	NS	Maintenance hemodialysis	3.13% citrate + 4 mg/mL gentamicin	Heparin 5,000 U/mL	20	20	2,002	1,734	NA	NA	58 ± 3	59 ± 4	CRBSI

CRBSI, Catheter-related bloodstream infection; CRI, catheter-related infection; ESI, exit-site infection; NA, not applicable; NS, not specified.

## Conclusions

Citrate lock solutions offer a dual advantage of anticoagulant and antimicrobial properties, effectively minimizing the risk of systemic coagulation dysfunction and bleeding. When combined with antibiotics, they emerge as a safe and highly effective alternative to heparin, demonstrating significant potential in reducing catheter-related complications. This makes citrate lock solutions a compelling choice for enhancing patient safety and optimizing clinical outcomes in catheter management.

## Data Availability

The raw data supporting the conclusions of this article will be made available by the authors, without undue reservation.
